# Letter in response to: “Randomised open label exploratory, safety and tolerability study with calmangafodipir in patients treated with the 12-h regimen of N acetylcysteine for paracetamol overdose—the PP100-01 for Overdose of Paracetamol (POP) trial: study protocol for a randomised controlled trial”

**DOI:** 10.1186/s13063-019-3476-3

**Published:** 2019-06-24

**Authors:** Jan Olof G. Karlsson, Per Jynge, Ingemar Lundström, Louis J. Ignarro

**Affiliations:** 1 Division of Drug Research, Department of Pharmacology, Linköping University, Linköping, Sweden; 2 Department of Physics, Chemistry and Biology, Linköping University, Linköping, Sweden; 3 UCLA School of Medicine, Beverly Hills, CA USA

As founders of PledPharma AB [1], including the main inventor of calmangafodipir [2], we have with great interest read the article entitled “Randomised open label exploratory, safety and tolerability study with calmangafodipir in patients treated with the 12-h regimen of N-acetylcysteine for paracetamol overdose—the PP100-01 for Overdose of Paracetamol (POP) trial: study protocol for a randomised controlled trial” published online by POP Trial Investigators and Dear [3] on January 8, 2019.

In the Background section of the article, POP Trial Investigators and Dear [3] state the following: “Calmangafodipir (Ca_4_Mn(DPDP)_5_) is a unique chemical species derived from mangafodipir, where 80% of the manganese in mangafodipir has been replaced with calcium. Based on the similarities between calmangafodipir and mangafodipir, it is anticipated that calmangafodipir would also exhibit SOD-dependent pharmacologic actions similar to those of mangafodipir”. We presume that they pertain the SOD-mimetic actions of these two compounds and *not* the SOD-dependent actions, as SOD is the admitted abbreviation of superoxide dismutases (intracellular and extracellular enzymes). Unfortunately, POP Trial Investigators and Dear have omitted an essential reference written by us and Torsten Almén (deceased January 8, 2016) [1]. This article describes the background of both calmangafodipir and mangafodipir, and their SOD-mimetic properties, so the word “anticipated” is highly misleading. The article by Brurok et al. [4] (reference 10 in POP Trial Investigators and Dear [3]) is interesting but it was published 13 years before calmangafodipir was described in a scientific journal [5]. You can find our article [1] on the sponsor’s website (https://www.pledpharma.com/pipeline/publications/), and as the title reads “Calmangafodipir [Ca_4_Mn(DPDP)_5_], mangafodipir (MnDPDP) and MnPLED with special reference to their SOD mimetic and therapeutic properties” there should be no doubt that this is the most appropriate reference to cite. The omission, whether deliberate or not, is a disservice to the reader. Furthermore, we believe that the earlier “original” work is actually the work that led to Dear’s clinical trial study.

## Data Availability

Not applicable.
